# PD-L1 expression in tumor tissue and peripheral blood of patients with oral squamous cell carcinoma

**DOI:** 10.18632/oncotarget.22576

**Published:** 2017-11-08

**Authors:** Manuel Weber, Falk Wehrhan, Christoph Baran, Abbas Agaimy, Maike Büttner-Herold, Raimund Preidl, Friedrich W. Neukam, Jutta Ries

**Affiliations:** ^1^ Department of Oral and Maxillofacial Surgery, Friedrich-Alexander University Erlangen-Nürnberg, Erlangen, Germany; ^2^ Institute of Pathology, Friedrich-Alexander University Erlangen-Nürnberg, Erlangen, Germany; ^3^ Institute of Pathology, Department of Nephropathology, Friedrich-Alexander University Erlangen-Nürnberg, Erlangen, Germany

**Keywords:** PD-L1, mRNA, PCR, OSCC, peripheral blood

## Abstract

**Background:**

Immune checkpoints like programmed cell death-1 (PD-1) and its ligand PD-L1 are involved in immune escape mechanisms of solid tumors including oral squamous cell carcinoma (OSCC). Inhibitors of the pathway are successfully used for treating especially advanced disease. However, the physiological relevance of PD-1/PD-L1-signaling in OSCC is insufficiently understood. The aim of the study was to analyze if PD-L1 expression in tumor tissue and peripheral blood samples of OSCC patients is associated with histomorphological tumor parameters and if PD-L1 expression in patients is different from controls.

**Results:**

OSCC tumor specimens showed a significantly higher PD-L1 expression than oral mucosa controls (*p* < 0.001; upregulation more than 3-fold). Cross-tabulation revealed an association of increased expression of PD-L1 mRNA in tissue specimens with malignancy (*p* < 0.001).

OSCC cases with higher tumor grade and cases with lymph node metastases (N+) were significantly (*p* < 0.05) associated with increased PD-L1 expression in peripheral blood. Cross-tabulation revealed an significant association with lymph node metastases (N+) (*p* ≤ 0.002).

**Materials and Methods:**

PD-L1 mRNA expression was analyzed in tumor specimens and corresponding samples of healthy oral mucosa and peripheral blood of 45 OSCC patients and 36 healthy control persons using RT-qPCR analysis. A Mann-Whitney *U-test*, a cut-off point analysis and a Chi-square test were carried out.

**Conclusions:**

PD-L1 expression in OSCC could contribute to the immunosuppressive local tumor microenvironment. Increased malignant behavior (higher tumor grade, positive nodal status) might be associated with PD-L1 mediated systemic immune tolerance. Thus, PD-L1 expression in peripheral blood might be an indicator of the existence of metastatic disease (N+) in OSCC.

## INTRODUCTION

There is emerging evidence that in addition to the biology of the tumor cell itself, the clinical course of malignant tumors is significantly influenced by the host immune response against cancer cells [1].

Although oral squamous cell carcinomas (OSCC) are highly immunogenic tumors [2], an effective host defense reaction is not observed. One reason is the presence of an immune tolerant tumor microenvironment [3, 4]. The current prognostic assessment of malignant tumors using the TNM-score does exclusively describe parameters of the neoplastic tissue and neglects the interaction between tumor and host [5]. Therefore, new complementary “immune-scoring” systems including host derived immune response and immune tolerance towards the neoplastic disease are needed [5].

Immune tolerance is mediated by inhibitory signaling pathways, so called immune checkpoints. The programmed cell death-1 (PD-1) and the PD-1 ligand (PD-L1) pathway is one of the most relevant immunologic checkpoints [6], that is involved in the immune escape mechanism of solid tumors like OSCC. PD-L1 binds to PD-1, a cell surface receptor expressed by activated T-cells and macrophages, and induces apoptosis of these immune cells [7] or formation of regulatory T-cells. Thus, overexpression of PD-L1 leads to immunosuppression [6, 8].

A constitutively high PD-L1 expression is seen in macrophages in immune privileged tissues like placenta and retina [6]. Besides immune cells, tumor cells can also express PD-L1 which might contribute to their immunosuppressive microenvironment [9].

An increased PD-L1 expression could already be shown in several solid malignancies like breast cancer, colon cancer or esophageal cancer [7]. Recent immunohistochemical studies indicate a possible connection between high PD-L1 expression in OSCC specimens and the occurrence of lymph node metastases [10, 11] and unfavorable prognosis [11–13]. These studies indicated that most but not all OSCC cases express PD-L1.

The emerging pharmacologic class of “checkpoint inhibitors” block suppressive immune signaling pathways like the PD-1/PD-L1 pathway. Current clinical studies investigating those “checkpoint inhibitors” for treatment of advanced clinical stages of solid malignancies revealed encouraging success rates that were not reached by any other second line treatment regime [14–16]. If patients respond to “checkpoint inhibitors”, the remission phases seem to be more durable than could be achieved by any other previous treatment [17] making “checkpoint inhibitors” the currently most promising approach for cancer immunotherapy.

In the meanwhile those therapies are standard treatment for advanced melanoma and non-small cell lung cancer [3]. In addition, there are several late phase clinical trials including patients with recurrent or metastatic head and neck squamous cell carcinomas (HNSCC) [18, 19]. In studies, targeting the PD-1/PD-L1 pathway by specific antibodies a significantly improved overall survival and quality of life could be shown [14]. Recently, inhibitors of the PD-1/PD-L1 pathway were approved in the US and the EU for advanced HNSCC refractory to platinum-based chemotherapy [20, 21]. Current protocols targeting the PD-1/PD-L1 pathway achieve response rates of up to 30% [22]. The fact that response rates do not necessarily correlate with the immunohistochemical detection of PD-L1 expression in tumor tissue [23, 24], outlines that the physiological relevance of PD/PD-L1 signaling is still insufficiently understood [23]. Hence, the clinical use of checkpoint therapies is more advanced than the fundamental biological understanding of immune regulation. For this reason, it is necessary to understand if PD-L1 mediated immune tolerance is a local phenomenon of the tumor microenvironment or a systemic condition. Thus, combined analysis of PD-L1 expression in tumor tissue and peripheral blood might be helpful. This analysis could contribute to an “immune-scoring” of OSCC that goes beyond TNM- classification and accommodates recent immune therapy strategies.

The current study was designed to clarify, if PD-L1 expression is altered in cancer tissue compared to healthy oral mucosal and in peripheral blood samples of OSCC patients in comparison to peripheral blood of healthy control persons. It was addressed, if a cut-off point (COP) can be determined allowing the differentiation of OSCC patients from healthy controls based on the PD-L1 expression in tissue or peripheral blood. Additionally, a possible association between PD-L1 expression in tissue or peripheral blood with histomorphological parameters (T-, N-, L-, Pn-status, tumor grade) was tested.

## RESULTS

### Clinical and histomorphological parameters of the analyzed cases

Tissue specimens (OSCC and heathy oral mucosa) and whole blood samples of 45 OSCC patients (group patients) and 36 healthy volunteers (group controls) were collected. Demographic characteristics of all participants and histomorphological parameters of all OSCC patients are documented in Table 1. 29 males and 16 females were included in the patients group. The control group consisted of 24 males and 12 females. Mean age was 64.6 years (SD 12.5) in the patients group and 57.7 years (SD 20.7) in the control group (Table 1).

**Table 1:** Description of the patient collective; total number of cases: 81

		patients		controls	
		*n*	% of cases	*n*	% of cases
**number of cases**		45		36	
**gender**	male	29	64.4	24	66.7
	female	16	35.6	12	33.3
**mean age**		64.6 years (SD 12.5)		57.7 years (SD 20.7)	
**age range**		35–93 years		15–88 years	
**T-status**	T1–T2	24	53.3		
	T3–T4	19	42.2		
	unknown	2	4.4		
**N-status**	N0	25	55.6		
	N+	18	40		
	unknown	2	4.4		
**L-status**	L0	32	71.1		
	L1	11	24.4		
	unknown	2	4.4		
**Pn-status**	Pn0	23	51.1		
	Pn1	20	44.4		
	unknown	2	4.4		
**grading**	G1	6	13.6		
	G2	27	60		
	G3	11	24.4		
	unknown	1	2.2		
**clinical stage**	early	16	35.6		
	late	27	60		
	unknown	2	4.4		

Demographic characteristics of OSCC patients (group patients) and healthy volunteers (group controls). For the OSCC patients group, staging parameters (T-, N-, L-, Pn-status, grading, clinical UICC stage) are shown.

### Comparison of PD-L1 expression in tissue and blood between OSCC patients and healthy volunteers

Data were derived from RT-qPCR and are presented as ΔCT values. PD-L1 expression in tissue samples was normally distributed. Shapiro-Wilk testing revealed a *p*-value of 0.365 for PD-L1_4 and 0.177 for PD-L1_2 in tissue specimens. In blood samples, PD-L1 expression was not normally distributed. Shapiro-Wilk testing revealed *p*-values < 0.001 for both PD-L1_4 and PD-L1_2 in blood samples. Using the Mann-Whitney U test, OSCC patients (group patients) and healthy controls (group controls) were tested for significant differences of mRNA expression for the transcript variants 1, 2 and 4 of PD-L1. In the analyses, expression of two isoforms of the gene was analyzed simultaneously (PD-L1 variants 1 and 2 named PD-L1_2 and PD-L1variants 1 and 4 named PD-L1_4) (Table 2). Higher ΔCT values indicate lower mRNA expression (Table 3).

**Table 2:** Real-time qPCR Primer

Primer	Sequence (5′ to 3′)	Primer (bp)	Amplicon (bp)	Annealing temperature (°C)
PD-L1_2^*^ s	AGACCACCACCACCAATTCC	20	173	60
PD-L1_2^*^ /as	TGGAGGATGTGCCAGAGGTA	20	–	–
PD-L1_4^**^ /s	AGCTATGGTGGTGCCGACTA	20	152	60
PD-L1_4^**^ /as	CAGATGACTTCGGCCTTGGG	20	–	–
GAPDH in /s	GACCCCTTCATTGACCTCAACTA	23	102	60
GAPDH in /as	GAATTTGCCATGGGTGGAAT	20	–	–

^*^amplification of transcript variant 1 and 2 simultaneously; amplicon named PD-L1_2

^**^amplification of transcript variant 1 and 4 simultaneously; amplicon named PD-L1_4

The selected primers for RT-qPCR mRNA expression analyses of PD-L1_4 (including PD-L1 splicing variants 1 and 4) and PD-L1_2 (including PD-L1 splicing variants 1 and 2).

**Table 3:** PD-L1 expression in tissue and peripheral blood of healthy controls and OSCC patients

	*n*	mean ΔCT	SD	*p*-value	AUC	FC	COP	No. of cases	+	-	% pos. cases	*p*-value χ^2^ test
**PD-L1_4 tissue**	78			< 0.001	0.83	3.19	7.99	78	41	37		< 0.001
controls	35	8.82	1.11					35	6	29	17.1%	
patients	43	7.15	1.59					43	35	8	81.4%	
**PD-L1_4 blood**	75			0.165	nd	nd	nd	nd	nd	nd	nd	nd
controls	31	7.17	1.40									
patients	44	6.90	1.24									
**PD-L1_2 tissue**	74			< 0.001	0.76	3.18	10.37	74	40	34		< 0.001
controls	31	10.92	1.45					31	8	23	25.8%	
patients	43	9.25	1.94					43	32	11	74.4%	
**PD-L1_2 blood**	75			0.287	nd	nd	nd	nd	nd	nd	nd	nd
controls	31	7.10	1.33									
patients	44	6.92	1.21									

Comparison of PD-L1 mRNA expression between OSCC patients (group patients) and healthy volunteers (group controls). Expression of PD-L1 splicing variants 1 and 4 (PD-L1_4) and splicing variants 1 and 2 (PD-L1_2) in tissue (OSCC tumor tissue vs. healthy oral mucosa of volunteers) and peripheral blood was analyzed. The mean ΔCT value (mean), standard deviation (SD) and the p-value provided by the Mann-Whitney *U* test are shown. Higher ΔCT values indicate lower PD-L1 mRNA expression. Regarding PD-L1 expression in tissue, area under the curve (AUC), fold-change FC and cut-off point (COP) values are given. Based on their ΔCT value related to the COP, the cases were determined as positive (malignant) and negative (healthy). The percentage of positive tested cases (% pos. cases) in the controls and patients group is presented. A Statistical analysis was carried out by the Chi-square test (χ^2^ test). Fold-change (FC) of PD-L1 mRNA expression was determined by the ΔΔCT method comparing the average ΔCT values of the two groups. nd: not determined.

**Figure 1:**
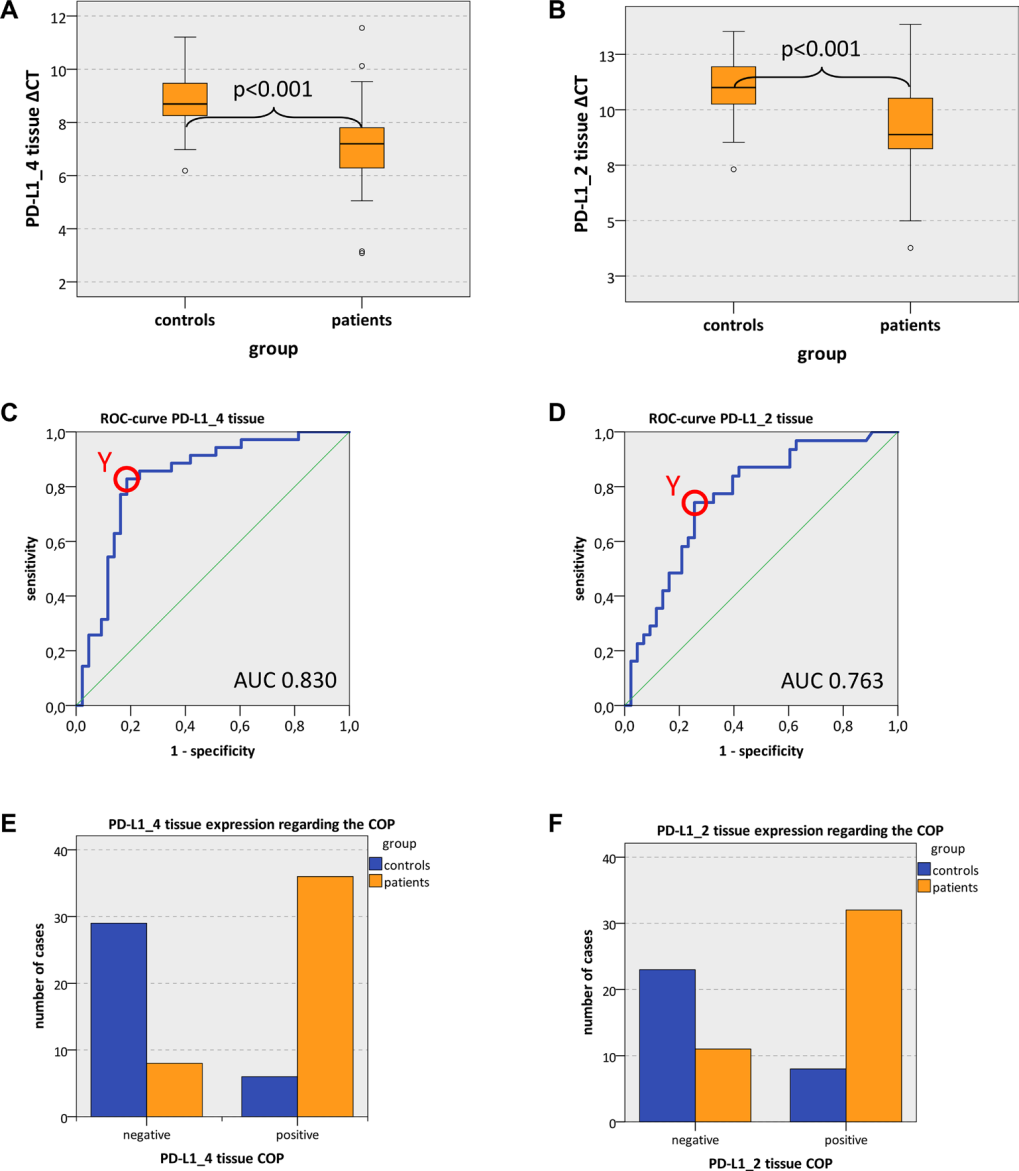
PD-L1 tissue expression in OSCC patients and healthy mucosal controls (**A**, **B**) Box plots of the median PD-L1 expression rates in tumor tissue of OSCC patients (group patients) and healthy oral mucosa of volunteers (group controls). The median ΔCT values of PD-L1 splicing variants 1 and 4 (PD-L1_4) (Figure 1A) and splicing variants 1 and 2 (PD-L1_2) (Figure 1B) derived from RT-qPCR are given. Higher ΔCT values indicate lower PD-L1 mRNA expression. The median, the interquartile range and the standard deviation are provided. Statistical analyses were carried out by the Mann-Whitney U test. (**C**, **D**) ROC curves for PD-L1 mRNA expression based on the RT-qPCR data. The diagrams are a plot of the sensitivity (true-positive rate) vs. 1-specificity (false-positive rate) over all possible ΔCT values. Data for PD-L1 variant PD-L1_4 (Figure 1C) and variant PD-L1_2 (Figure 1D) are provided. The circles show the points of the highest Youden (Y) indices which are associated with the COP (patients vs. controls). The AUC value is indicated. ROC: receiver operating characteristic, COP: cut-off point, AUC: area under the curve.e, (**F**) Division of the test and control group (group patients and group controls) into positive and negative subgroups based on the ascertained COPs of PD-L1 variant PD-L1_4 (Figure 1E) and variant PD-L1_2 (Figure 1F) expressed as ΔCT values. Using the χ^2^ test, the specimens were positively (malignant) judged if the values lied below the COP. Increased PD-L1_4 and PD-L1_2 expression levels in the tissue of OSCC patients (group patients) compared healthy oral mucosa of volunteers (group controls) were significant. Therefore, the COP may be a parameter allowing the allocation of a tissue sample to a group and the proof of malignancy.

Both analyzed PD-L1 variants (PD-L1_4 and PD-L1_2) showed significantly increased expression in OSCC compared to normal oral mucosa (mean PD-L1_4 ΔCT patients 7.15, mean PD-L1_4 ΔCT controls 8.82; *p* = 0.001; mean PD-L1_2 ΔCT patients 9.25, mean PD-L1_2 ΔCT controls 10.92; *p* < 0.001) (Table 3, Figure 1A, 1B). These values indicate a significant 3.19-fold upregulation of PD-L1_4 and a significant 3.18-fold upregulation of PD-L1_2 expression in OSCC compared to oral mucosa (Table 3). Hence, the biological active isoforms of PD-L1 were over expressed in OSCC.

In order to confirm the statistical relevance, ROC curves (Figure 1C and 1D) were generated and the AUC was determined. The upregulated PD-L1_4 yielded an AUC of 0.83 (Table 3, Figure 1C) and the upregulated PD-L1_2 reached an AUC of 0.76 (Table 3, Figure 1D). Thus, this analysis confirmed that both PD-L1 mRNA variants were of significant diagnostic value for discrimination between healthy volunteers and OSCC patients.

The highest Youden indices were 0.643 for PD-L1_4 and 0.486 for PD-L1_2 (Figure 1C and 1D). The optimal threshold values (COPs) expressed in ΔCT standards for distinguishing the patients from the healthy controls were 7.99 for PD-L1_4 and 10.37 for PD-L1_2 (Table 3). For all PD-L1 mRNA splice variants (PD-L1_4 and PD-L1_2), a ΔCT value lower than the COP (upregulated PD-L1 expression) was considered to be positive for malignancy. Using the determined COPs, the two groups (patients and controls) were divided into positive and negative specimens in order to confirm that these parameters allow the detection of malignancy in a certain sample. The statistical evaluation by the Chi-square test revealed that the increased expression rates of PD-L1 mRNA were statistically relevantly associated with malignancy. The results are summarized in Table 3 and illustrated in Figure 1E and 1F.

Out of the OSCC patients, 81.4% (35/43) showed increased PD-L1_4 expression and 74.4% (32/43) exhibited increased PD-L1_2 expression. In contrast, only 17.1% and 25.8% of the control samples (normal mucosa) showed increased PD-L1_4 and PD-L1_2 expression, respectively (Table 3, Figure 1E and 1F). The correlation of malignancy and the detection of increased PD-L1 expression rates in tissue specimens were significant for both PD-L1 mRNA variants (*p* < 0.001) (Table 3). Thus, increased expression of PD-L1 mRNA in tissue specimens was significantly associated with malignancy and may indicate the existence of OSCC. A sensitivity of 81.4% and a specificity of 82.9% were determined for PD-L1_4 expression. For PD-L1_2 a sensitivity of 74.4% and a specificity of 74.2% were calculated (Table 5). Data for positive- and negative predictive value of PD-L1 expression for diagnosis of malignancy are given in Table 5.

**Table 4:** PD-L1 expression in peripheral blood of OSCC patients related to histomorphological parameters (T-, N-, L-, Pn-status, grading)

		n	mean ∆CT	SD	*p*-value	AUC	FC	COP	No. of cases	+	-	% pos. cases	*p*-value χ^2^ test
PD-L1_4 tissue		78			< 0.001	0.83	3.19	7.99	78	41	37		< 0.001
controls		35	8.82	1.11					35	6	29	17.1%	
patients		43	7.15	1.59					43	35	8	81.4%	
PD-L1_4 blood		75			0.165	nd	nd	nd	nd	nd	nd	nd	nd
controls		31	7.17	1.40									
patients		44	6.90	1.24									
PD-L1_2 tissue		74			< 0.001	0.76	3.18	10.37	74	40	34		< 0.001
controls		31	10.92	1.45					31	8	23	25.8%	
patients		43	9.25	1.94					43	32	11	74.4%	
PD-L1_2 blood		75			0.287	nd	nd	nd	nd	nd	nd	nd	nd
controls		31	7.10	1.33									
patients		44	6.92	1.21									

Association between the PD-L1 mRNA expression rates in peripheral blood samples of OSCC patients and histomorphological parameters (T-, N-, L-, Pn-status, grading). Data for PD-L1 splicing variants 1 and 4 (PD-L1_4) and splicing variants 1 and 2 (PD-L1_2) are given. The mean ΔCT value (mean), standard deviation (SD) and the p-value provided by the Mann-Whitney U test are shown. Higher ΔCT values indicate lower PD-L1 mRNA expression. Regarding the N-status, area under the curve (AUC), fold-change FC and cut-off point (COP) values are given. Based on their ΔCT value related to the COP, the cases were determined as positive (+) and negative (-). The percentage of positive tested cases (% pos. cases) in the N0 and N+ group is presented. A Statistical analysis was carried out by the Chi-square test (χ^2^ test). Fold-change (FC) of PD-L1 mRNA expression was determined by the ΔΔCT method comparing the average ΔCT values of the two groups. nd: not determined.

**Table 5:** Sensitivity, specificity, positive- and negative predictive value of PD-L1 expression for diagnosis of malignancy and N-status

		+	-	sensitivity	specificity	positive predictive value	negative predictive value
	**PD-L1_4 tissue**						
**Diagnose**	controls	6	29				
(tissue)	patients	35	8	81.4%	82.9%	0.854	0.784
	**PD-L1_2 tissue**						
	controls	8	23				
	patients	32	11	74.4%	74.2%	0.8	0.68
	**PD-L1_4 blood**						
**N-status**	N0	11	13				
(blood)	N+	17	1	94.4 %	54.2 %	0.607	0.929
	**PD-L1_2 blood**						
	N0	8	16				
	N+	15	3	83.3%	66.7%	0.652	0.842

The table shows sensitivity, specificity, positive- and negative predictive value of PD-L1 expression in tissue specimens for diagnosis of malignancy. (controls vs. patients) and of PD-L1 expression in blood samples for diagnosis of a positive N-status (N0 vs. N+).

In contrast to the described results in tissue specimens, there was no significant difference in PD-L1 mRNA expression with regard to blood samples of OSCC patients and healthy controls (PD-L1_4: mean ΔCT patients 6.90, mean ΔCT controls 7.17; *p* = 0.165; PD-L1_2: mean ΔCT patients 6.92, mean ΔCT controls 7.10; *p* = 0.287) (Table 3).

### Association of PD-L1 expression in tissue and blood samples with histomorphological parameters (T-, N-, L-, Pn-status, grading) of OSCC patients

Using Mann-Whitney U test, comparison of PD-L1 mRNA expression in tissue specimens (OSCC tumor and normal oral mucosa) regarding histomorphological parameters (T-, N-, L-, Pn-status, grading) revealed no significant associations. Higher ΔCT values indicate lower mRNA expression (Table 4).

**Figure 2:**
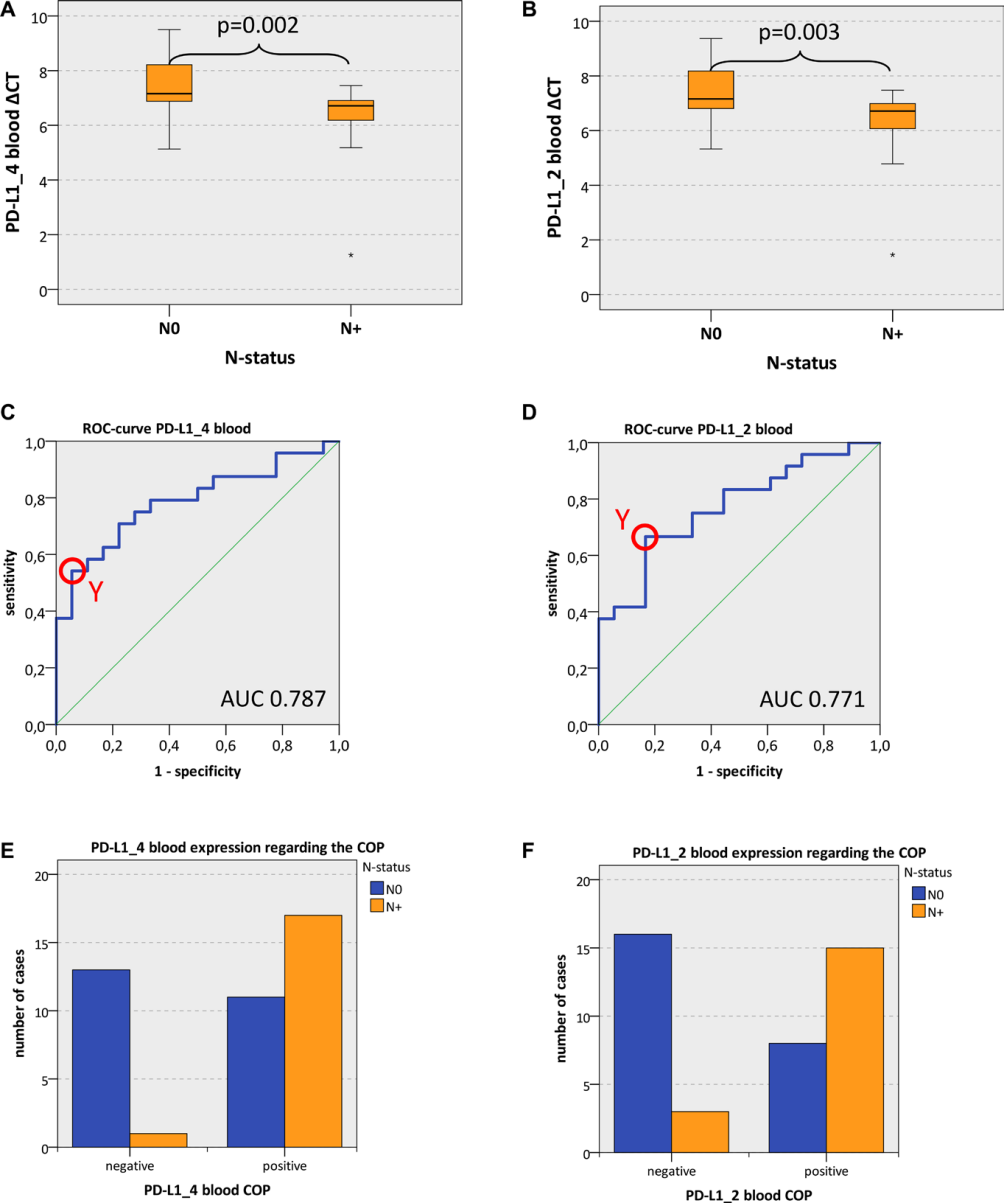
PD-L1 blood expression in OSCC patients depending on the N-status (**A**, **B**) Box plots of the median PD-L1 expression rates in peripheral blood of OSCC patients with lymph node metastases (group N+) and OSCC patients without lymph node metastases (group N0). The median ΔCT values of PD-L1 splicing variants 1 and 4 (PD-L1_4) (Figure 2A) and splicing variants 1 and 2 (PD-L1_2) (Figure 2B) derived from RT-qPCR are given. Higher ΔCT values indicate lower PD-L1 mRNA expression. The median, the interquartile range and the standard deviation are provided. Statistical analyses were carried out by the Mann-Whitney *U* test. (**C**, **D**) ROC curves for PD-L1 mRNA expression based on the RT-qPCR data. The diagrams are a plot of the sensitivity (true-positive rate) vs. 1-specificity (false-positive rate) over all possible ΔCT values. Data for PD-L1 variant PD-L1_4 (Figure 2C) and variant PD-L1_2 (Figure 2D) are provided. The circle shows the point of the highest Youden (Y) index which is associated with the COP (N+ vs. N0). The AUC value is indicated. ROC: receiver operating characteristic, COP: cut-off point, AUC: area under the curve. (**E**, **F**) Division of the test and control group (N+ OSCC cases and N0 OSCC cases) into positive and negative subgroups based on the ascertained COPs of PD-L1 variant PD-L1_4 (Figure 2E) and variant PD-L1_2 (Figure 2F) expressed as ΔCT values. Using the χ^2^ test, the specimens were positively (N+) judged if the values lied below the COP. Increased PD-L1_4 and PD-L1_2 expression levels in the peripheral blood of OSCC patients with lymph node metastases (group N+) compared to OSCC patients without lymph node metastases (group N0) were significant. Therefore, the COP may be a parameter allowing the allocation of a blood sample to a case with (group N+) or without (group N0) lymph node metastases.

Expression of both analyzed PD-L1 variants (PD-L1_4 and PD-L1_2) in whole blood samples of OSCC patients was significantly higher in cases with lymph node metastases (N+) compared to cases without lymph node metastases (N0) (mean PD-L1_4 ΔCT N+ 6.29, mean PD-L1_4 ΔCT N0 7.35; *p* = 0.002; mean PD-L1_2 ΔCT N+ 6.30, mean PD-L1_2 ΔCT N0 7.37; *p* = 0.003) (Table 4, Figure 2A, 2B). These values indicate a significant 2.09-fold upregulation of PD-L1_4 expression and a significant 2.1-fold upregulation of PD-L1_2 expression in peripheral blood of N+ OSCC patients compared to N0 patients (Table 4).

In order to confirm the statistical relevance, ROC curves (Figure 2C and 2D) were generated and the AUC was determined. Upregulated PD-L1_4 mRNA yielded an AUC of 0.79 (Table 4, Figure 2C) and upregulated PD-L1_2 mRNA reached an AUC of 0.77 (Table 4, Figure 2D). Thus, this analysis confirmed that both PD-L1 mRNA variants were of significant diagnostic value for discrimination of N+ and N0 OSCC patients using peripheral blood samples. The results showed a statistically significant association between PD-L1 overexpression and the prevalence of lymph node metastases (N+).

The highest Youden indices were 0.486 for PD-L1_4 and 0.5 for PD-L1_2 (Figure 2C and 2D). The optimal threshold values (COPs) expressed in ΔCT standards for distinguishing the N+ patients from N0 patients were 7.14 for PD-L1_4 and 7.04 for PD-L1_2. For both PD-L1 mRNA variants (PD-L1_4 and PD-L1_2), a ΔCT under the COP (upregulated) was considered to be positive for lymph node metastases (N+) corresponding to an increased expression level in peripheral blood samples. Using the determined COPs, the two groups (N+ and N0 patients) were divided into positive and negative lesions in order to confirm that PD-L1 expression in blood allowed the detection of lymph node metastases (N+ status). The statistical evaluation by the Chi-square test revealed that increased expression rates of PD-L1 mRNA in peripheral blood were significantly associated with the presence of lymph node metastases (N+ status). The results are summarized in Table 4 and illustrated in Figure 2E and 2F. Regarding the diagnosis of positive N-status (N+), a sensitivity of 94.4% and a specificity of 54.2% were determined for PD-L1_4 expression in blood samples. For PD-L1_2 a sensitivity of 83.3% and a specificity of 66.7% were calculated (Table 5). Data for positive- and negative predictive value of PD-L1 expression in blood samples for diagnosis of positive N-status (N+) are given in Table 5.

**Figure 3:**
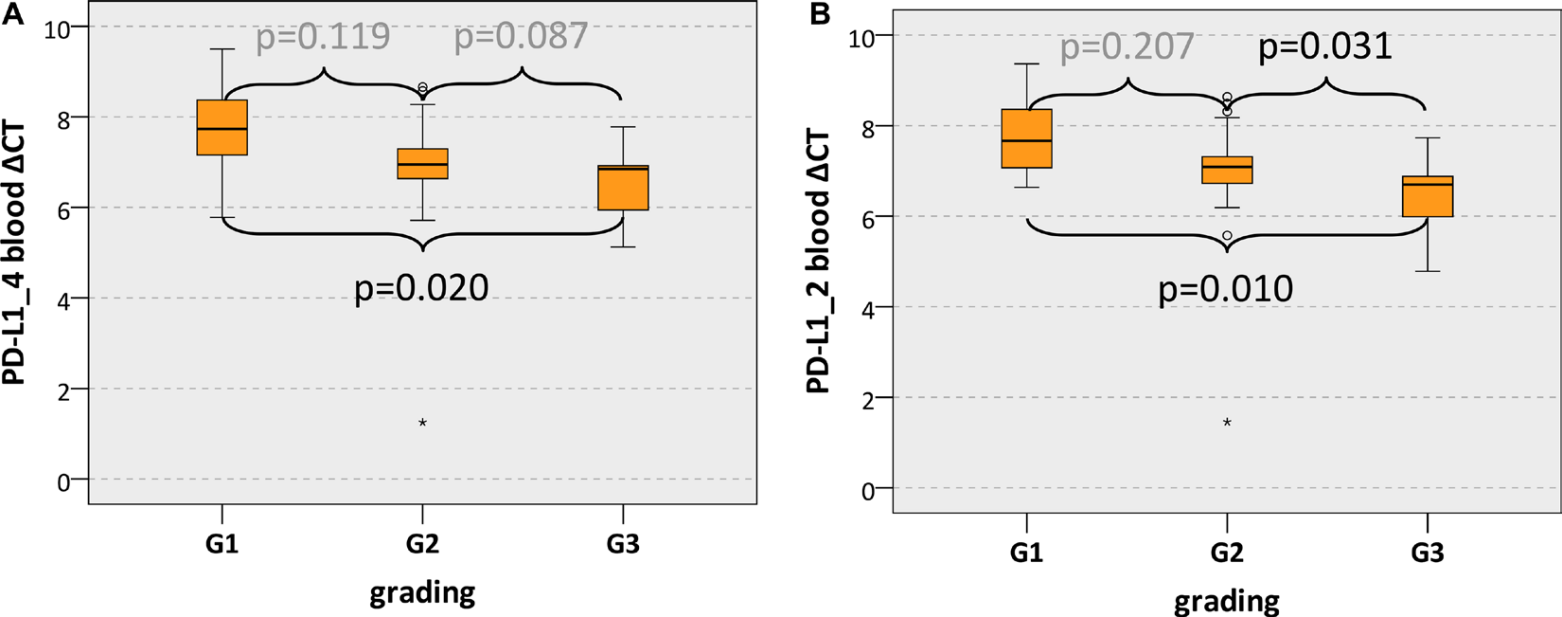
PD-L1 blood expression in OSCC patients depending on the grading Box plots of the median PD-L1 expression rates in peripheral blood of OSCC patients depending on the tumor grading (G1-G3) The median ΔCT values of PD-L1 splicing variants 1 and 4 (PD-L1_4) (Figure 3A) and splicing variants 1 and 2 (PD-L1_2) (Figure 3B) derived from RT-qPCR are given. Higher ΔCT values indicate lower PD-L1 mRNA expression. The median, the interquartile range and the standard deviation are provided. Statistical analyses were carried out by the Mann-Whitney *U* test.

Additionally, there was a significant association between PD-L1 mRNA expression in blood samples and the histologically determined tumor grading. PD-L1_4 expression in blood samples of G3 cases was significantly higher than in samples of G1 cases (mean PD-L1_4 ΔCT: G3 6.47, G1 7.71; *p* = 0.020) (Table 4, Figure 3A). PD-L1_2 expression in blood of G3 cases was significantly higher than in G1 cases (*p* = 0.010) and in G2 cases (*p* = 0.031) (mean PD-L1_2 ΔCT: G3 6.42, G2 6.93, G1 7.79) (Table 4, Figure 3B).

There was no significant association between the altered blood expression of PD-L1 mRNA and tumor size (T1/T2 vs. T3/T4) (*p* > 0.05). Moreover, no significance was shown with regard to changes in blood expression rates of PD-L1 mRNA and histologically proven lymph vessel infiltration (L-status) or perineural infiltration (Pn-status). The results of statistical assessment are summarized in Table 4.

## DISCUSSION

### PD-L1 expression in healthy individuals and OSCC patients

The current study revealed an increased PD-L1 mRNA expression in OSCC tumor tissue compared to healthy oral mucosa. PD-L1 signaling leads to an inhibition of T-cell activation and proliferation [16] which promotes the immune escape of the tumor. The detected increase of PD-L1 expression in OSCC tissue might originate from tumor cells as well as from tumor infiltrating immune cells and can be an indicator of the tumor-induced local immunosuppressive microenvironment.

It is assumed that tumor cells upregulate PD-L1 expression to evade the host immune reaction and thereby to increase their survival rate [28]. PD-L1 expression in tumor cells is induced intrinsically by oncogenic signaling pathways and extrinsically by factors of the tumor micro-environment [23, 28]. Activation of the oncogenic MAPK signaling pathway was shown to be an intrinsic activator of PD-L1 expression [23, 29]. Tumor hypoxia is one extrinsic driver of PD-L1 upregulation. Increased levels of the hypoxia-associated transcription factor Hif1α were shown to be associated with high PD-L1 expression [23]. Additionally, different growth factors [23] as well as cytokines [28] can modulate PD-L1 expression of tumor cells. The results of the current study indicate that PD-L1 expression increases at a certain point or period during malign transformation of oral mucosa to OSCC.

We detected PD-L1 mRNA expression in all analyzed OSCC specimens and in all samples of control mucosa tissue. PD-L1 expression in OSCC was previously investigated in several immunohistochemical studies [10–13, 28, 30–32]. These studies report rates of PD-L1 positive cases from 18% to 87% [28]. The heterogeneity of these results might be explained by the use of different staining protocols and the subjective assigning of cut-off points to determine PD-L1 positivity [18, 28]. To our knowledge, this is the first study determining PD-L1 mRNA expression in OSCC specimens compared to healthy controls. Moreover, a cut-off point was determined at which an overexpression of PD-L1 can be detected objectively.

Immunohistochemically identified PD-L1 positivity is often used as potential predictor for therapy response of PD-L1 or PD-1 targeting checkpoint inhibitors [28]. However, there is a large proportion of clinical cases with immunohistochemically positive tumor cells that do not respond to checkpoint inhibitor therapy [23, 24]. Genetic alterations associated with weakened or changed antigen presentation might lead to resistance to checkpoint inhibitors in some of these cases [33, 34]. On the contrary, there are tumors that do not express PD-L1 on the cell surface and respond to antibodies targeting the PD/PD-L1 pathway [23, 24]. Immunocompetent mice bearing PD-L1 knockout tumors showed a significant treatment response to PD-L1 blocking antibodies with a long-term survival of nearly 90% [35]. Additionally, a recent flow cytometric analysis showed an increased expression of PD-L1 in tumor infiltrating lymphocytes in HNSCC [36]. These findings indicate that PD-L1 expression on immune cells might be of equal or of even higher biological relevance than on tumor cells [35, 37].

### Correlation of PD-L1 expression with histomorphological parameters in OSCC patients

So far, there are no studies investigating PD-L1 expression in peripheral blood of tumor patients. In the current analysis, all tested peripheral blood samples of OSCC patients and healthy controls showed positive PD-L1 expression. Generally, PD-L1 is expressed on T-cells, dendritic cells and monocytes/macrophages [3, 19]. Therefore, it is conceivable that these types of immune cells express PD-L1 in whole blood samples and that this expression could be altered in OSCC patients compared to healthy volunteers and additionally in patients suffering from different clinical stages of OSCC. In the here applied method all blood components were included in the expression analyses. PD-L1 expression of circulating tumor cells is therefore overlain by immune cell based PD-L1 expression [38–40]. Hence, changes in the immune response and possible systemic immune suppressive mechanisms in tumor progression could be assessed by PD-L1 expression analysis. Therefore, in the current analyses of blood samples, immune cell based PD-L1 expression is expected to be more relevant than PD-L1 expression on disseminated tumor cells. However, in order to confirm this hypothesis further studies are required in which individual cell fractions of peripheral blood are isolated, characterized and examined for their PD-L1 expression. In the current study a significantly increased PD-L1 expression in the peripheral blood of OSCC patients with lymph node metastases (N+) was detected. Local PD-L1 expression is caused by tumor cells, healthy epithelial cells or inflammatory cells. Systemic PD-L1 expression detected in peripheral blood is presumably caused in large parts by circulating immune cells. Therefore, the detected association between lymph node metastases and high PD-L1 expression in peripheral blood of OSCC patients is an indicator that PD-L1 expression of immune cells might be of high biologic relevance.

As PD-L1 signaling leads to immunosuppression [37], the increased PD-L1 expression in blood samples of N+ cases detected in the current study indicates that metastatic disease in OSCC patients might be associated with a PD-L1 mediated systemic state of immunosuppression. It is unclear if this is caused by the presence of metastatic tumor tissue or if a systemic state of PD-L1 mediated immune tolerance enables the formation of metastatic lesions in tumor draining lymph nodes. The fact that OSCC patients and healthy control persons do not differ regarding their PD-L1 expression in peripheral blood indicates that a tumor-independent, preexisting state of immune surveillance or PD-L1 mediated immune tolerance might enable the formation of metastases in a certain subgroup of individuals. Thus, the systemic expression of PD-L1 might be an indicator of the competence – or incompetence – of the host organism to deal with the tumor.

The current study revealed no significant correlation between PD-L1 expression in OSCC tumor specimens and histomorphological parameters. PD-L1 expression in tumor tissue is caused by tumor cells and by tumor infiltrating immune cells. Thus, in OSCC tumor samples, effects of different tumor cell derived PD-L1 expression could overlay the immune cell based association between N-status and PD-L1 expression that was detected in peripheral blood samples.

Macrophages are a possible source of PD-L1 expression in peripheral blood [37]. Previous studies showed an association between immune tolerant M2-polarized macrophages and high PD-L1 expression in cancer specimens [41–43]. In the current analysis, we could detect an association between higher tumor grading and increased PD-L1 expression in peripheral blood samples. This finding could indicate that increased malign potential of the primary tumor correlates with an augmented degree of peripheral immune tolerance. In peripheral blood, OSCC patients and controls showed no difference in the number of CD14/CD16 expressing cells representing both M1- and M2-polarized macrophages [44]. However, we identified an association between markers of malignant behavior (grading, L-status, Pn-status) in the primary tumor with a shift of macrophage polarization in regional lymph nodes from the anti-tumoral M1 polarization towards the tumor-promoting M2 polarization in a previous study [45]. As M2 polarization of macrophages is also considered as an expression of immune tolerance [46] and M2 macrophages are characterized by PD-L1 expression [41, 42], it can be hypothesized that increased malignancy of the primary OSCC is associated with augmented peripheral immune tolerance. This hypothesis is supported by a recent *in vitro* analysis showing an increased PD-L1 expression in macrophages and dendritic cells co-cultured with high grade OSCC cells [3]. The relevance for OSCC is underlined by a further study showing increased PD-L1 expression in CD163 and CD204 positive M2 macrophages derived from OSCC patients *in vitro* [47].

### Limitations of the study

As mRNA expression was analyzed, post transcriptional modifications might lead to an altered PD-L1 protein expression.

The current study cannot determine to which extent the observed increase in PD-L1 expression in OSCC specimens is caused by tumor cells or by stromal cells and tumor-infiltrating immune cells. However, the increased PD-L1 expression in blood samples (PAXgene) of N+ OSCC cases seems to represent a condition of the host immune system. There is currently conflicting data regarding a positive or inverse correlation between PD-L1 expression and the infiltration of lymphocytes in OSCC [28, 30, 31].

### Future perspectives

It is not clear at which point during the tumorigenesis of OSCC PD-L1 expression is upregulated [23]. Therefore, analysis of PD-L1 expression in precursor lesions of OSCC like oral leukoplakia and squamous dysplasia would be of great interest.

The question if PD-L1 expression on tumor cells or immune cells like macrophages is of higher relevance for the tumor immune-escape mechanism is not yet answered. Therefore, immunohistochemical analysis of the association between PD-L1 expression and macrophage polarization would be of interest.

Preclinical data indicate that a combination of PD-L1 inhibition and radiotherapy is an effective treatment regime for OSCC [48]. Therefore, an analysis of PD-L1 expression in peripheral blood of OSCC patients before and after radiotherapy might give new insights to motivate future treatment studies.

Finally, the results of the current report could motivate to test the potential role of PD-L1 expression in blood as predictive biomarker for the presence of metastatic disease (N+) in OSCC patients in prospective studies.

## MATERIALS AND METHODS

### Patients and sample collection

The study was approved by the Ethics Committee of the University of Erlangen-Nuremberg, Erlangen, Germany (approval number: 3962) and patients’ written informed consent was obtained. The study was performed in accordance with the Declaration of Helsinki.

Tissue specimens and peripheral blood samples were collected from 45 OSCC patients (group patients) and 36 healthy volunteers (group controls). Patients were only included in this study if they presented with OSCC for the first time. All samples were taken before any treatment (i.e. radiotherapy and/or chemotherapy). Demographic characteristics including age and gender of all study participants were documented and are shown in Table 1. Healthy volunteers were selected based on the absence of inflammation and malignant disease. Tissue samples of healthy volunteers were taken during dentoalveolar surgery after informed consent.

The diagnosis of OSCC was assigned through routine histopathological examination. Grading (G1-G3; differentiation status), clinical UICC-stage (I–IV) and TNM classification of OSCC were documented in the histologic reports according to the guidelines of the most recent World Health Organization classification of tumors of the head and neck and the International Union Against Cancer [25, 26]. Clinical stages were grouped as early (stage I and II) and late (stage III and IV) stages. Additionally, lymph node status was grouped as N0 and N+ to indicate absence (N0) or presence (N+) of metastases, respectively. Additionally, subgroups were categorized based on tumor size and/or invasion dividing the samples into small/early (T1 and T2) and large/advanced (T3 and T4) malignancies. Relevant clinical and histopathological parameters are summarized in Table 1.

Sampling of tumor specimens and whole peripheral blood

Tissue samples of healthy volunteers were obtained during minor surgery by avoiding additional incisions. In case of OSCC patients, tumor tissue samples were acquired during tumor resection. In order to conserve mRNAs after excision all tissue specimens were immediately transferred into RNAlater (Qiagen, Hilden, Germany) and were fixed by incubation at 4°C for at least 24 hours. Afterwards they were stored at -80°C until mRNA isolation.

Additionally, two samples of 2.5 ml whole peripheral blood were collected in a PAXgene Blood RNA Tube (PreAnalytiX GmbH, Hombrechtikon, Switzerland) from healthy volunteers as well as from OSCC patients before tumor removal. The samples were carefully inverted 8–10 times, incubated at room temperature for two hours and stored at -20°C for 24 hours. Storage up to RNA isolation was carried out at -80°C.

### Isolation of mRNA and RT-qPCR analysis

Whole RNA was extracted using miRNeasy mini-Kit (Qiagen, Hilden, Germany) for tissue samples and the PAXgeneBlood miRNA Kit (PreAnalytiX GmbH, Hombrechtikon, Switzerland) for blood samples. Subsequently, the RNA samples were stored at -80°C until expression analysis by RT-qPCR analyses.

### Real-time quantitative reverse transcription-PCR (RT-qPCR) analysis

The total-RNA was transcribed into cDNA using the Transcriptor High-Fidelity cDNA Synthesis Kit according to the manufacturer`s recommendations (Roche, Mannheim, Germany). For quantification of PD-L1 expression gene-specific primer for three isoforms (transcript variants 1, 2 and 4) of the gene were used (Table 2). The PD-L1 gene has four splice variants. Variants 1, 2 and 4 are biological active and were analyzed in the current study. Splice variant 3 of PD-L1, a pseudogene was not analyzed, as it is not translated. Two splice variants (PD-L1 variant 1 and 2 named amplicon PD-L1_2 and PD-L1 variant 1 and 4 named amplicon PD-L1_4) were simultaneously amplified in each PCR-reaction (Table 2). For amplification, the QuantiTect SYBR^®^ Green PCR Kit (Qiagen) was applied. Data acquisition and analysis was performed using the ABI Prism 7300 of Applied Biosystems (ThermoFisher Scientific Inc., Waltham, MA, USA). The values of RT-qPCR analyses were normalized by the ΔCT method using GAPDH as internal control. The relative quantification of differences in gene expression between the two groups was based on the cycle threshold method (ΔΔCT-method, RQ = 2-ΔΔCT) using Microsoft Excel 2016.

### Statistical analysis of RT-qPCR

For statistical evaluation of the RT-qPCR analyses, the program IBM SPPS Statistics 22 (Chicago, IL, USA) was applied. The average of duplicate ΔCT values of each sample was used for the data results. Expression data were controlled for normal distribution by Shapiro-Wilk test. Furthermore, the average values of all ΔCT within a group were calculated and used to determine relative quantification (RQ) of the examined genes between the two groups by the ΔΔCT method. Twofold changes in mRNA expression rates (2 ≤ RQ ≤ 0.5) were defined as statistically relevant. Statistical relevance of the apparent expression between the two groups was analyzed by Mann-Whitney U -test. P -values ≤ 0.05 were considered to indicate a statistically significant result. Based on ΔCTvalues graphical diagrams are plotted as Box-Whisker plots which represent the median, the interquartile range, standard deviation, minimum and maximum values of mRNA expression.

In order to assess the discriminatory accuracy of the marker for distinguishing between two groups, receiver operator characteristic (ROC) curves were created using the expression profile of each differentially expressed mRNA. The value for the area under the ROC curve (AUC) defines the usefulness of a mRNA with respect to its ability to separate the tumor tissue samples from those of healthy volunteers. Additionally, by using the ROC curve the highest Youden index was determined. This value is associated with the threshold value, also named “cut-off-point” (COP) for the biological marker. The COP indicates which value of decreased or increased expression is relevant for the discrimination between malignant and normal samples and allows assigning a particular sample to a certain group [27].

Based on these COPs, the two groups were divided into two subgroups which showed an expression rate over or under the COP. Afterwards, associations between altered mRNA expression, malignancy and histomorphological parameters were calculated by the Chi-square test (χ^2^ test).

## CONCLUSIONS

PD-L1 expression in OSCC tumor tissue was significantly higher than in healthy oral mucosa. PD-L1 expression in the peripheral blood of OSCC patients was associated with high tumor grading and metastatic disease (N+). This indicates an association between more aggressive behavior of the tumor and a systemic state of immune tolerance.

In future studies, PD-L1 expression should also be analyzed in precursor lesions of OSCC to clarify the role of PD-L1 signaling during early stages of malignant transformation. Such studies might pave the way for establishing novel diagnostic markers that would reliably predict the risk of malignant transformation of oral leukoplakia to OSCC and thus enable the use of immune modulatory therapy concepts already in early stages of OSCC tumorigenesis.
